# Application of Neural Network with Autocorrelation in Long-Term Forecasting of Systemic Financial Risk

**DOI:** 10.1155/2022/7131143

**Published:** 2022-09-16

**Authors:** Junzhi Zhang, Lei Chen

**Affiliations:** School of Management, China University of Mining and Technology-Beijing, Beijing 100083, China

## Abstract

Carrying out early warning of systemic financial risk is a prerequisite for timely adjustment of monetary policy and macroprudential policy to effectively prevent and resolve systemic financial risks. This paper constructs a systemic financial risk monitoring and early warning system for China's banking industry based on isolated forest anomaly detection and neural network with autocorrelation mechanism and uses low-frequency data with high credibility to effectively identify the ten factors that have the greatest impact on systemic financial risk in China's banking industry, improving the prospective and accuracy of risk early warning. The conclusions can help regulators to adjust their policies prospectively to curb the rise of systemic financial risks.

## 1. Introduction

The subprime crisis in 2007 made governments and international academics realize that microprudential regulation for individual financial institutions insufficiently reflects the actual risk accumulation in the financial system and that macroprudential regulation for financial system needs to be strengthened to maintain financial stability. In the following decade or so, governments have devoted themselves to the establishment and improvement of macroprudential policy frameworks. Among them, early warning (EW) of systemic financial risk (SFR) is crucial in macroprudential supervision. On December 31, 2021, People's Bank of China (PBC) formulated and published Macroprudential Policy Guidelines (Trial Implementation), which emphasize the need to establish a sound framework for SFR's monitoring, assessment, and early warning and actively explore the use of big data technology in SFS's monitoring and early warning. In China's Monetary Policy Implementation Report (2022 Q1), PBC pointed out that financial work should further improve the macroprudential policy framework, improve SFR's monitoring, assessment, and early warning capabilities, and enrich macroprudential policy toolbox. Thus, it is clear that exploring the construction of SFR's EW system will be a long-term topic in financial research.

Regarding the EW models of SFR, the commonly used models include the logit model [1–3], probit model [4], KLR signal model [5], STV cross-sectional regression model [6], and so on. In addition, many scholars have also combined the analytical frameworks such as binary classification tree (BCT) [7], GARCH model [8], quantile projection [9], and jump undetermined equity analysis [10] to further improve the prediction models of systemic financial risk. Further, with the continuous research in this field, the latest research has made good progress in effectively predicting systemic financial risk using cutting-edge machine learning models such as random forest [11–13], neural network [12, 14–16], and support vector machine (SVM) [12, 17, 18].

At present, domestic research studies on systemic financial risk early warning are also quite abundant [19–23], but they also face the following difficulties: (1) the use of high-frequency data, such as stock data, can improve the timeliness of risk early warning. However, relevant studies also point out that the development of China's financial market is relatively short and immature, and the effectiveness of public market data needs to be further tested [24, 25]; (2) the risk early warning system using asset and liability data can better reflect the risk changes in related domestic fields, but the low frequency of data limits the foresight of early warning; and (3) risk forecasting techniques based on econometric models, such as GARCH-like models, are to fit in each sequence separately, which cannot take advantage of the similarity patterns existing in different sequences.

The development of deep learning provides a useful exploration to solve the above difficulties. The autoformer model is improved on the basis of transformer based on deep decomposition architecture and the autocorrelation mechanism [26]. The model has improved the efficiency of long-term prediction through progressive decomposition and sequence-level connection and also makes the model itself capable of making long-term time series prediction based on less data.

Motivated by the above analysis, we construct a SFR's EW system for China's banking industry around the unique characteristics of the autoformer model. The reason for taking the banking industry as the early warning research object is that the banking industry in China accounts for more than 90% of the total assets of the financial industry for a long time, and the stability of banks is crucial to financial stability.

This paper is displayed as follows: firstly, we review the influencing factors of SFS and organize the set of covariate indicators for EW of bank systemic financial risk (BSFR) indexes in a phase dependent manner; secondly, we adopt the variance threshold method for initial screening of indicators and propose an indicator selection method based on the isolated forest method for further screening of indicators, the purpose of which is to downscale indicators and screen out indicators with high correlation with BSFR, and lay the foundation for the monitoring and EW of BSFR; thirdly, the early warning model is constructed around autoformer to predict the changes of bank systemic risk. Finally, this research is summarized, and conclusions and recommendations are drawn.

The contributions are as follows: (1) The indicator screening method based on isolated forest anomaly detection is completely data-driven, which not only identifies several factors that have the greatest impact on SFR of China's banking industry but also alleviates the interference of human subjective factors to a certain extent and (2) constructing an EW system for SFR based on autoformer not only exploits the high credibility of low-frequency data but also achieves the forward-looking nature of early warning.

## 2. Materials and Methods

### 2.1. Covariate Set Selection

#### 2.1.1. Theoretical Analysis of SFR's Influencing Factors

Systemic financial risk refers to the risk caused by internal factors such as financial vulnerability or external factors such as policy adjustments and macroeconomic fluctuations, which can spread through the interinstitutional correlation network and cause the dysfunction of financial institutions, thus leading to the dysfunction of financial services, the spread of market panic, and ultimately leading to serious damage to the macro economy.

Based on the above definition of SFR and actuality of China's financial system, we grouped the factors into five dimensions:

Macroeconomic risk: Macroeconomic and financial systems have a clear “pro-cyclicality.” When the macro economy continues to improve, credit in the banking sector rises rapidly and credit standards are relaxed; when the economy is in a downward phase, the downward pressure is transmitted to the banking sector, and the banks' asset quality comes under pressure, and credit contracts and the NPL rate rises. In the process of economic downturn and bank credit contraction, the default of the real economy induces liquidity crisis and credit crisis in the banking sector.

Credit risk: Against the backdrop of a continuous decline in macroeconomic growth, some enterprises' operating efficiency and debt-servicing ability have declined significantly, highlighting credit risk in the banking industry and deteriorating credit asset quality. As credit risk intensifies, it will lead to large-scale interest defaults, deterioration of bank-enterprise relationship, and mutual trust in the near term; in the long run, it will weaken the profitability of banks, and capital will be continuously eroded by nonperforming loans, and banks will be forced to contract credit when it is more difficult to replenish capital from external sources, resulting in a vicious circle of “credit contraction—contraction of the real economy—credit contraction.”

Market risk: Declines in interest rates, equity, and foreign exchange and commodity prices can cause loss in banks' balance sheet operations. Currently, the capital finance lenders of equity financing and bond financing in China's capital market are mainly commercial banks, and the fall in the value of stocks and bonds erodes the quality of banks' assets and affects their normal operations. In addition, commercial banks' credit assets include mostly real estate collateral, and a decline in housing prices will lead to significant losses in bank assets.

Liquidity risk: Liquidity risk is the possibility that a bank will not have sufficient liquidity to settle liabilities as they fall due and meet customer withdrawal needs without increasing costs or losing asset value, resulting in a loss to the bank. The current liquidity risk of banks in China mainly comes from the business model of “borrowing short and lending long.”

Correlation risk: Interbank correlation amplifies the contagion capacity of various risks. Under the modern credit-money system, monetary funds intended to maintain systemic stability and lower market interest rates may intensify commercial banks' over-reliance on wholesale funds and make profits through arbitrage by amplifying leverage and increasing maturity mismatch, thus generally forming a nested and intertwined interbank credit network, where problems in one institution will quickly spread to the periphery, increasing the demand for liquidity settlement and exacerbating systemic risk.

#### 2.1.2. Selection of Factors Influencing Systemic Financial Risk

Based on the above theoretical analysis and previous researches [13, 18, 27–31], this paper selects covariate indicators for constructing the systemic financial risk early warning in the banking sector system. The indicators of macroeconomic risk include GDP growth rate (GDP), M2 growth rate (M2), consumer confidence index (CCI), growth rate of total retail sales of consumer goods (TRSCG), consumer price index (CPI), product inventory growth rate of industrial enterprises (PIIE), fixed assets investment growth rate (FAI), industrial entrepreneur confidence index (IECI), industrial enterprise prosperity index (IEPI), investment growth in urban real estate development (IURED), state housing boom index (SHB), government leverage ratio (GLR), leverage ratio of resident sector (RLR), and leverage ratio of real economy department (RELR). The indicators of credit risk include commercial bank provision coverage rate (PCR) and nonperforming loan ratio of commercial banks (NPL). The indicators of liquidity risk include weighted average interest rate of interbank lending (WAIR), capital adequacy ratio of commercial banks (CAR), liquidity ratio of commercial banks (LRCB), loan-to-deposit ratio of commercial banks (LDR), and commercial bank excess reserve ratio (ERR). The indicators of market risk include exchange rate (ER), CSI corporate bond index (CBI), CSI 300 index (CSI 300), proportion of real estate loans (REL), and proportion of foreign exchange loans (FEL). The indicators of correlation risk include interbank asset dependence (IBA) and interbank liability dependence (IBL).

Since no systemic financial crisis has occurred in China, it is not appropriate to use 0 or 1 as a proxy for the change of systemic financial risk. In view of this, this section adopts the Chinese systemic financial risk (SRISK) data published by the Stern School of Business of New York University as a proxy variable for systemic financial risk, and the covariate data are obtained from the websites of National Bureau of Statistics, PBC, CBRC, and Wind. The time span is from February 2011 to December 2020.

### 2.2. Screening of Covariate Indicators

Commonly used indicator selection methods are filtering, wrapping, and embedding methods. Except for some algorithms in the filtering method, other algorithms are generally used for supervised learning, while the risk identification problem studied in this section is not easy to get the category to which the sample belongs in advance and should belong to unsupervised learning [29].

Based on the above analysis, this section adopts an indicator screening method that includes the variance threshold method and isolated forest method [29]. Isolated forests segment samples by random features and anomalies are more easily isolated compared to ordinary sample points so that anomalous sample points have shorter paths. Therefore, whether a sample point is an outlier can be determined by the following equation:

(1) $$\begin{array}{c} S\left(x,\psi \right)=2^{-\left(E\left(h\left(x\right)\right)/c\left(\psi \right)\right)}\text{,} \end{array}$$

where *h*(*x*)is the height of sample point *x* in each tree, *c*(*ψ*) is the average of the path lengths for a given number of samples *ψ,* and *S* (*xψ*) denotes the anomaly score value of sample point *x*. If the anomaly score *S* is close to 1, then the sample point *x* must be an anomaly; if the anomaly score *S* is much less than 0.5, then the sample point *x* must not be an anomaly.

The specific steps of indicator screening combining the variance threshold method and isolated forest anomaly detection are as follows:(1) Find the variance of all indicators and use the mean of variance as the threshold to initially screen the indicator set. (2) Randomly select a subset of the indicator set obtained in the first step and standardize the indicator data of the subset. (3) Use isomap to downscale the standardized subset of indicator data. The isomap algorithm can better control the loss of data information and can represent the data in higher dimensions more comprehensively in the lower dimensional space. (4) Apply the isolated forest method to the downscaled data for anomaly detection and obtain the score of each sample point. (5) Repeat step (4) 10 times to obtain the average score of each sample point. (6) Obtain the Euclidean distance from each abnormal sample point to the normal sample centre in the subset and obtain the median of all Euclidean distances in the corresponding subset. (7) Repeat steps (2) to (6) *n* times to obtain *n* median Euclidean distances, and the subset with the largest median Euclidean distance is the best subset.

### 2.3. Systemic Financial Risk Early Warning Model

For time series forecasting problems, traditional statistical approaches focus on providing parametric models from the domain expertise level, such as autoregressive (AR), exponential smoothing, or structural time series models, but the application of traditional statistical methods is also limited by data validity and frequency. Autoformer models based on deep decomposition architecture provide a new research solution to address these issues. It can significantly improve long-time forecasting by coping with complex temporal patterns and information utilization bottlenecks through progressive decomposition and sequence-level connectivity.

Autoformer completely revolutionizes transformer as deep decomposition architecture, embedding sequence decomposition into the encoder-decoder as an internal unit of autoformer [26]. In the prediction process, the model alternately optimizes the prediction results and decomposes the sequence; that is, the trend item and the periodic item are gradually separated from the hidden variables to realize the gradual decomposition.

The sequence decomposition unit is based on the idea of moving average, smoothing the period term, and highlighting the trend term:

(2) $$\begin{array}{c} x_{t}=AvgPool\left(Padding\left(x\right)\right)\text{,}\\ x_{s}=x-x_{t}\text{,} \end{array}$$

where *x* is the hidden variable to be decomposed, and *x*_*t*_ and *x*_*s*_ are the trend term and the period term, respectively. The above equations are collectively referred to as the series decomposition *x*_*t*_, *x*_*s*_=*SeriesDecomp*(*x*) and will be embedded into autoformer layers.

In the encoder part, the model gradually eliminates the trend term and obtains the periodic terms *S*_*en*_^*l*,1^, *S*_*en*_^*l*,2^. Based on this periodicity, the model aggregates similar subprocesses of different periods by designing an autocorrelation mechanism to achieve information aggregation. The relevant equations are as follows：

(3) $$\begin{array}{c} S_{en}^{l,1},=SeriesDecomp\left(AutoCorrelation\left(x_{en}^{l-1}\right)+s_{en}^{l-1}\right)\text{,}\\ S_{en}^{l,2},=SeriesDecomp\left(Fee\;dF\;orwar\;d\left(S_{en}^{l,1}\right)+S_{en}^{l,1}\right)\text{.} \end{array}$$

In the decoder part, the model shows the trend term and the period term separately, in which, for the period term, the autocorrelation mechanism uses the periodic nature of the sequence to aggregate subsequence with similar processes in different cycles; for the trend term, the trend information is gradually extracted from the predicted hidden variables using a cumulative approach. The correlation equation is as follows:

(4) $$\begin{array}{c} S_{de}^{l,1},T_{de}^{l,1}=SeriesDecomp\left(AutoCorrelation\left(x_{de}^{l-1}\right)+x_{de}^{l-1}\right)\text{,}\\ S_{de}^{l,2},T_{de}^{l,2}=SeriesDecomp\left(AutoCorrelation\left(x_{de}^{l,1},x_{en}^{N}\right)+S_{de}^{l,1}\right)\text{,}\\ S_{de}^{l,3},T_{de}^{l,3}=SeriesDecomp\left(Fee\;dF\;orwar\;d\left(S_{de}^{l,2}\right)+S_{de}^{l,2}\right)\text{,}\\ T_{de}^{l}=T_{de}^{l-1}+W_{l,1}*T_{de}^{l,1}+W_{l,2}*T_{de}^{l,2}+W_{l,3}*T_{de}^{l,3}\text{.} \end{array}$$

Based on the above progressive decomposition architecture, the autoformer model can gradually decompose the hidden variables in the forecasting process and obtain the forecasting results of periodic and trend components through autocorrelation mechanism and accumulation, respectively, so as to realize the alternate and mutual promotion of decomposition and prediction results optimization.

Based on the above analysis, this paper adopts autoformer to construct the early warning system of China's banking systemic financial risk. The early warning model takes systemic financial risk as the variable to be predicted and the best subset of indicators is screened based on the variance threshold method and isolated forest algorithm as covariates.

## 3. Results

### 3.1. Covariates Screening and Analysis

#### 3.1.1. Result of the Variance Threshold Method

The principle of the variance threshold method is to first estimate the variance of each indicator and then screen the indicators according to a threshold value. If the variance of an indicator is small, it means that the overall fluctuation of the indicator is small; that is, it contributes less to the anomaly, and the indicator can be removed more safely. The calculation steps are as follows: (1) standardize the sample data; (2) calculate the variance of each indicator; and (3) filter the indicators according to a threshold value. The obtained calculation results are displayed in Table 1.

The mean value of variance (0.06679) is treated as threshold, and 15 indicators are selected for the systemic financial risk early warning model: commercial bank provision coverage rate (PCR), nonperforming loan ratio of commercial banks (NPL), leverage ratio of resident sector (RLR), interbank asset dependence (IBA), consumer confidence index (CCI), CSI corporate bond index (CBI), leverage ratio of real economy department (RELR), loan-to-deposit ratio of commercial banks (LDR), exchange rate (ER), liquidity ratio of commercial banks (LRCB), M2 growth rate (M2), state housing boom index (SHB), interbank liability dependence (IBL), government leverage ratio (GLR), and CSI 300 index.

#### 3.1.2. Result of Isolated Forest

Isolated forest anomaly detection is applied for further screening indicators. All subsets are selected using the exhaustive method, and the median Euclidean distance of each abnormal sample point is calculated for all subsets. The subset of indicators corresponding to the largest median Euclidean distance includes commercial bank provision coverage rate (PCR), nonperforming loan ratio of commercial banks (NPL), leverage ratio of resident sector (RLR), interbank asset dependence (IBA), consumer confidence index (CCI), CSI corporate bond index (CBI), loan-to-deposit ratio of commercial banks (LDR), liquidity ratio of commercial banks (LRCB), state housing boom index (SHB), and interbank liability dependence (IBL). The top five subsets with the largest median Euclidean distances are shown in Table 2.

Table 3 shows the definition of the selected indicators. Consumer confidence index, leverage ratio of residential sector, and state housing boom index belong to macroeconomic risk; CSI corporate bond index belongs to market risk; nonperforming loan ratio and provision coverage ratio of commercial banks belong to credit risk of commercial banks; loan-to-deposit ratio and liquidity ratio reflect liquidity risk of commercial banks; and interbank asset and liability dependence reflects the correlation risk of commercial banks.

#### 3.1.3. Logical Analysis of the Selected Indicators and Systemic Financial Risk

The indicator screening system based on the variance threshold method and the isolated forest anomaly detection method provides the selected indicators for the systemic financial risk early warning system in a purely data-driven manner, but the monetary policy and macroprudential supervision policy for preventing, controlling, and resolving systemic financial risks need to clarify the drivers behind the risks in order to accurately block and defuse risks. Therefore, this subsection analyses the logical relationship between the selected indicators and systemic financial risk from the perspective of the correlation between modern money supply and bank liquidity.

Under the modern credit monetary system, the central bank issues the base currency through asset expansion, and commercial banks create broad money through asset expansion [32]. In general, a broad money supply system with the base currency as the reserve is formed. Under this monetary supply system, the central bank injects liquidity into commercial banks by expanding and adjusting assets, and commercial banks provide liquidity to the banking system and the real economy through asset-side creation and active debt behaviour. Under the modern credit monetary system, China has now formed the following liquidity transmission structure: the central bank injects liquidity into commercial banks through debit or open market operations (liquidity facilitation tools); commercial banks with primary traders receive funds first, and then can lend them to small and medium-sized banks that do not have the qualifications of primary traders through interbank certificates of deposit and lending; commercial banks put credit into three areas such as government platform, real estate, and nonbank financial institutions; and the liquidity of nonbank financial institutions also flows to government platform and real estate.

Based on the above analysis, the logical relationship between the selected indicators and China's systemic financial risk is as follows: expectations about the economy (consumer confidence index) influence the leveraging behaviour of the residential sector (residential sector leverage ratio), which in turn influences commercial banks' liquidity allocation to the real estate market and the real economy (State Housing Boom Index and CSI Corporate Bond Index); commercial banks' liquidity allocation behaviour influences their credit risk (nonperforming loan ratio and provision coverage ratio) and liquidity risk (loan-to-deposit ratio, liquidity ratio). Under the external disturbance, large commercial banks replenish their liquidity gap through wholesale funding, while small and medium-sized banks rely on interbank business (interbank assets and interbank liabilities dependency) to obtain liquidity. Under the influence of residents' leveraging behaviour and commercial banks' liquidity placement and acquisition behaviour, China's systemic financial risks show a clustering and accumulation situation.

### 3.2. Systemic Financial Risk Forecasting and Analysis

#### 3.2.1. Model Prediction Based on the Predictor and Covariates Indicators

The prediction indicators and covariate indicators are input into the autoformer model. In modelling, the data from February 2011 to December 2018 are divided into training sets, the data from January 2019 to December 2019 are divided into verification sets, and the data from January 2020 to December 2020 are used as test sets. The prediction error (MSE, MAE) of January 2020 (1 step), March 2020 (3 steps), June 2020 (6 steps), September 2020 (9 steps) is used to measure the model test error. The smaller the value of MSE and MAE, the better the prediction effect of the model.

Autoformer was used to predict SFR, and the prediction results are displayed in Table 4. For comparative analysis, this subsection also uses transformer and LSTM to predict SFR, where LSTM is used as the baseline model.

As shown in Table 5, the autoformer model predicts the systemic financial risk much better than the other two models. The steady change of autoformer as the prediction length increases indicates that autoformer has long-term robustness.

#### 3.2.2. Model Prediction Based on the Predictor

Early warning models are mostly constructed based on traditional econometrics, such as ARIMA and GARCH. The input of those models is only the predictor. Thus, we also only input the predictor into autoformer.

The results of the univariate prediction evaluation based on the predictor are shown in Table 5. The autoformer model still achieves better results in the long-term prediction.

## 4. Conclusion

Forward-looking early warning of systemic financial risks is necessary to ensure the sound operation of the banking system, which in turn lays the foundation for the safety and stability of the financial system.

This paper adopts the isolated forest algorithm and autoformer model to construct a systemic financial risk early warning system, which mainly solves the following problems: (1) Providing a theoretical basis for the selection of machine learning indicators. Based on the theoretical analysis of the mechanism of systemic financial risk generation in banks, this paper collates 28 indicators from five dimensions such as macroeconomic risk, market risk, liquidity risk, credit risk, and correlation risk, and then screens a total of 10 systemic financial risk early warning covariate indicators based on the variance threshold method and isolated forest algorithm, and analyses the logical relationship between the selected indicators and systemic financial risk. (2) Improving the accuracy of long-run forecasting of systemic financial risks. Compared with other machine learning models and traditional econometric models, this paper improves the prediction accuracy of systemic financial risk based on the autoformer model and also maintains robustness in long-order time prediction, which provides a better reference for regulatory policy expectation management.

This paper argues that in order to effectively prevent systemic financial risks, regulators can consider relying on administrative means to regularly obtain data on changes in the total amount of business transactions among financial institutions in addition to focussing on liquidity and credit risks of financial institutions so as to facilitate the analysis of correlation changes among financial institutions. In addition, according to the documents published by the regulators, the current additional regulation for systemically important banks in China mainly provides requirements in terms of additional capital, leverage ratio, large risk exposure, corporate governance, recovery and disposal plan, information disclosure and data reporting, etc., and involves less on liquidity management. Therefore, regulators should appropriately increase the content of liquidity management, develop liquidity management tools or indicators, and strengthen liquidity supervision of systemically important banks.

## Figures and Tables

**Table 1:** Variance of indicators.

Indicator	Variance	Indicator	Variance

PCR	0.13569	CSI300	0.06702
NPL	0.12835	CAR	0.06413
RLR	0.11398	REL	0.06120
IBA	0.09969	PIIE	0.05430
CCI	0.09319	ERR	0.04928
CBI	0.09303	FEL	0.04746
RELR	0.09038	CPI	0.03768
LDR	0.08995	IECI	0.03276
ER	0.08632	IEPI	0.03062
LRCB	0.08626	IURED	0.02932
M2	0.08441	WAIR	0.02409
SHB	0.07815	FAI	0.02285
IBL	0.07685	TRSCG	0.01378
GLR	0.06792	GDP	0.01138

**Table 2:** The five largest subsets of Euclidean distance.

Index	Subset	Distance

1	PCR, NPL, RLR, IBA, CCI, CBI, LDR, LRCB, SHB, IBL	1.668514
2	PCR, NPL, RLR, IBA, CCI, CBI, LRCB, SHB, IBL	1.649928
3	PCR, NPL, IBA, CCI, CBI, ER, LRCB, SHB, IBL	1.622538
4	PCR, NPL, IBA, CCI, CBI, RELR, LDR, M2, IIBL	1.604764
5	PCR, NPL, RLR, IBA, CBI, RELR, LDR, LRCB, SHB, IBL, GLR	1.599431

**Table 3:** Definition of selected indicators.

Indicator	Definition

Consumer confidence index	Reflects consumers' expectations to overall economic development and the consumer sector

Leverage ratio of residential sector	It is the ratio of residents' debt to disposable income and an important indicator of the level of residents' debt

State housing boom index	A comprehensive index reflecting the boom status of the real estate industry

CSI corporate bond index	A comprehensive index reflecting the overall price movement trend of corporate bonds in the exchange market

Commercial bank provision coverage rate	Reflects bank's financial stability and loss absorption capacity

Nonperforming loan ratio of commercial banks	Reflects the safety of the bank's credit assets

Loan-to-deposit ratio of commercial banks	The ratio of total bank loans to total deposits

Liquidity ratio of commercial banks	The ratio of liquid asset to liquid liability, which measures the overall level of commercial banks' liquidity

Interbank asset dependence	Ratio of interbank assets to total assets

Interbank liability dependence	Ratio of interbank liabilities to total liabilities

**Table 4:** Prediction results based on the predictor.

Model		Autoformer		Transformer		DeepAR		LSTM		ARIMA	

Metrics		MSE	MAE	MSE	MSE	MAE	MAE	MSE	MAE	MSE	MAE

Prediction length	1	**0.0498**	**0.1883**	0.0702	0.2166	0.0885	0.2262	0.2561	0.4354	0.1903	0.3630
	3	**0.0503**	**0.1946**	0.0782	0.2260	0.0976	0.2525	0.2599	0.4269	0.2076	0.3360
	6	**0.0527**	**0.1991**	0.1019	0.2651	0.1734	0.3230	0.2293	0.4074	0.2207	0.3391
	9	**0.0850**	**0.2468**	0.1778	0.3228	0.2263	0.3796	0.4464	0.5174	0.2797	0.4176

**Table 5:** Prediction results based on the predictor and covariates indicators.

Model		Autoformer		Transformer		LSTM	

Metrics		MSE	MAE	MSE	MAE	MSE	MAE

Prediction length	1	**0.1997**	**0.3047**	0.7443	0.6600	2.0166	1.0742
	3	**0.2230**	**0.2417**	0.9108	0.7229	2.2230	1.0677
	6	**0.2744**	**0.2718**	1.2039	0.7880	2.4971	1.0996
	9	**0.3496**	**0.3739**	3.0996	1.3835	2.6220	1.2000

## Data Availability

The data used to support the findings of this study are available from the corresponding author upon request.
